# Forty Years after Poverty Reduction in China: The Role of Women’s Empowerment in Enhancing Food Security and Diet Diversity

**DOI:** 10.3390/nu15122761

**Published:** 2023-06-15

**Authors:** Yanfang Huang, Fengying Nie, Xiangping Jia

**Affiliations:** Agricultural Information Institute, Chinese Academy of Agricultural Sciences, Beijing 100081, China; huangyanfang01@caas.cn (Y.H.);

**Keywords:** gender, nutrition and health, agricultural production diversity, agrifood transformation, food security

## Abstract

This study analyzed the role of women’s empowerment in diversifying the diet of households through their own production. Developed from empowerment and food security theories, this study constructed measures from the household dietary diversity score (HDDS) and Women’s Empowerment Index (WEI). The study focused on poverty-stricken regions in China and conducted a thematic questionnaire-based household survey on gender and food consumption in 2021. Based on micro-level evidence from 1199 rural households, this research found a low score of women’s empowerment and an average WEI of 0.689; the status of diet diversity, measured by the HDDS, varied by income and social class; and the average rate was low. Agricultural production diversity and women’s empowerment are positively associated with diet diversity. There is strong evidence that women’s employment mitigates the side effects of decreasing production diversity on households’ diet security. As a result, women’s empowerment can potentially mitigate the adverse consequences of low agricultural diversification in household diet quality in less developed areas. This study provides evidence for repurposing food and agricultural policies toward healthy diets and gender-responsive agrifood systems.

## 1. Introduction

Global concerns and advocacy over food security and healthy diets are rising. According to the United Nations, 702 to 828 million people in the world faced chronic undernourishment in 2021, and the hungry population may be more than 670 million in projections [1]. When moderate and severe food insecurity combined were counted, around 2.3 billion people in the world lacked access to adequate food in 2021, and almost 3.1 billion people could not afford a healthy diet due to increased costs in 2020 [1]. It has been widely recognized that a low level of diet diversity is detrimental to transforming agrifood systems toward higher nutritional values [2].

The role of women in ensuring rural food security varies widely by region and is changing rapidly. Globally, women comprised, on average, 36% of the agricultural labor force in developing countries in 2019. This figure represents a decline of about 7% since 2009, with a higher level of 66% in Sub-Saharan Africa and 71% in southern Asia in 2019 [2,3]. The gap in food insecurity between men and women widened from 1.7 percentage points in 2019 to 4.3 percentage points in 2021, mainly because of the COVID-19 pandemic [1]. If half of the small-scale producers in these areas had received development interventions that focused on empowering women, the income and food security of an additional 58 million people would have been assured. In addition, there would have been an increased resilience to shocks and stresses, such as pandemic and climate change, for an additional 235 million people [3]. Increasing women’s empowerment is essential for well-being and has a positive impact on food security, diet, and child nutrition [1].

In *The State of Food and Agriculture 2010–2011*, gender was focused more on inequalities in terms of access to resources and achievements [2]. Consequently, women’s empowerment has become popular and widely used to frame stated objectives related to agriculture and food systems. The narratives of gender-based agrifood systems began to take a more holistic view through the established framework of women’s agency and the developed tools of the Women’s Empowerment in Agriculture Index (WEAI). In spite of efforts to advocate for the use of these frameworks in monitoring national programs of agricultural and food policies, to our knowledge, the adoptions and adaptations of women’s empowerment remain anecdotal and piecemeal when designing and evaluating agrifood policies, especially in China.

Recently, policies on production measures and their impacts on food consumption decisions have been debated [4]. On the one hand, studies have shown that households’ decision making on agricultural production diversity determines diet diversity [5], which positively affects the food security and nutrition of household members [6,7,8,9,10]. However, on the other hand, some scholars have questioned the strategy of improving diet diversity through agricultural production diversity [11,12]. They argue that agricultural production diversity is not the major factor influencing diet diversity [13], with other factors having a greater impact, including income [14,15], market access [16,17,18], and urbanization [19]. The association between households’ decision making in agricultural production and diet diversity and exploring the moderating effects of women’s empowerment remains unclear.

A number of prior studies have attempted to investigate the connection between women’s empowerment and food security. These studies found that women’s empowerment impacts household welfare and food security [20,21,22,23,24]. However, to our knowledge, the gender patterns of production decision making are rarely integrated when studying dietary diversity and directing policy interventions in agrifood system transformation toward a healthy diet. Importantly, the integration of diet diversity and gender patterns reflects the rethinking of food attributes, such as calories, without looking at alternative characteristics, such as micronutrients and environmental sustainability.

The study focuses on China’s less developed areas that were identified and targeted by the national campaigns for poverty reduction. Over the past 40 years, the number of people in China with incomes below 1.90 USD per day has fallen by close to 800 million, accounting for three-quarters of global poverty reduction since 1980 [25]. In the 2011 *Outline for Development-Oriented Poverty Reduction for China’s Rural Areas*, a total of 832 poverty-stricken counties were identified and targeted for poverty alleviation. This campaign was highlighted as one of the top priorities, and massive political and economic resources were directed toward it [26]. However, low diversity in food consumption (dietary) remains a major problem for smallholder farmers in the region and affects their welfare. Therefore, our study aims to bring fresh insights into the relationship between production and consumption through a gender perspective within the Chinese context.

## 2. Conceptual Framework

### 2.1. Food Security and Diet Diversity

Diet diversity is an important indicator of household food security and well-being. Household food security is an important dimension of well-being. The inability of households to access enough food for a healthy and active life is a contributor to and outcome of their poverty. We followed the U.S. Agency for International Development (USAID) concept of food security; namely, all people at all times should have access to safe and nutritious food sources to maintain a healthy and productive life [27]. There are three dimensions to this definition of food security: availability (a measure of the adequacy of food supply and production), accessibility (a measure of whether a population has enough income to acquire food in the market), and utilization (a measure of the population’s ability to benefit from sufficient nutrition). Therefore, it is necessary to develop an appropriate measure of food security.

A number of approaches have been developed to measure diet diversity at the household level. Dietary diversity indicators (DDIs) have been used as a measurement tool for food and nutritional security, particularly in developing countries [28]. Recently, significant progress has been made in developing and validating DDIs with consistent and relevant meaning across different contexts and over time [29,30,31]. Four types of DDIs have been identified [32], including “food item-based indicators” (FIIs), “food group-based indicators” (FGIs), “dietary guidelines-based indicators” (DGIs), and “other indicators” (OIs). FIIs are based on the number of different food items consumed over a given period [33,34,35]. FGIs are based on the number of different food groups consumed over a reference period while allocating the same weighting to food groups [36,37,38]. DGIs are based on the number of different food groups consumed over a given period and are allocated different weightings according to dietary guidelines [39,40,41,42]. The OIs include mainly the Simpson Index [43,44], the entropy index [45], and the QUANTIDD index [46].

Counting foods or food groups through the food diversity score (FVS) and dietary diversity score (DDS) has been a popular measure of dietary diversity. The FVS estimates the number of different food items consumed over the past 24 h [47]. The DDS is a dietary quality index proposed by the Food and Agriculture Organization of the United Nations (FAO). The household dietary diversity score (HDDS) is a food security index at the household level that is computed on the basis of food consumption data over a past 24 h period. Using this approach, food can be categorized into twelve groups, i.e., cereals, potatoes, vegetables, fruits, meat, eggs, fish and seafood, milk, legumes, fats, sugars, and condiments. The index takes values by summing up the food group scores above. Following the Food and Nutrition Technical Assistance (FANTA) proposal, the value of the HDDS was divided into three groups, and the mean of the HDDS in the highest group was set as the target value to assess the household diet security [48].

### 2.2. Agency and Women’s Empowerment

We largely draw on Kabeer’s empowerment framework for our analysis. Kabeer [49] defines power as an individual’s ability to make and exercise choice, which incorporates three interrelated domains to the process of empowerment: resources (material and non-material), agency, and achievements. Agency does not only encompass observable actions but also the motivation for these actions and the meanings given to them. We also include under agency, one’s ability to influence through positions and identities. For instance, in some societies, an elder (a respected older person with power) can influence the actions or behaviors of their family members solely by their position and identity, without any verbal actions or decisions.

Empowerment is central to the global development agenda and agrifood transformations. In the Sustainable Development Goals of the United Nations, gender is identified and reflected in multiple targets for SDG 5: Achieve Gender Equity and Empower All Women and Girls [50]. Since The State of Food and Agriculture 2010–2011 was published by the United Nations Food and Agricultural Organization [2], gender was oriented and widely used in the stated objectives of agriculture and food systems. At the Food Systems Summit, organized by the United Nations General Assembly on 23 September 2001, gender was targeted as a critical element in the transformative change in agrifood systems.

The concept of agency underpins the development of the Women’s Empowerment in Agriculture Index (WEAI). Prior to the first launch of the WEAI in 2012, there was no metric that focused exclusively on measuring women’s agency in the agrifood sector [51]. The original structure of the WEAI included ten indicators across five equally weighted domains, i.e., decisions about agricultural production, access to and decision-making power about productive resources, control over the use of income, leadership in the community, and time allocation for working and leisure. On the basis of these, a number of adaptations were further developed at the project level (called pro-WEAI) by reframing women’s empowerment into intrinsic, instrumental, and collective agency [52]. The flexibility of optional add-on modules of the WEAI appealed to the interests of projects, leading to the scaled-up use of these tools. By 2022, it was estimated that the WEAI was used across 58 countries and 243 organizations [53]. As a measurement of agency, the WEAI supports better project designs and allows for monitoring and assessment, which further builds accountability and credibility in projects and policies [54,55].

### 2.3. Linkages between Production, Empowerment, and Nutrition

Agricultural diversity may directly influence food and nutritional security in rural households. Households consume a large share of the food products they produce, and more diverse product portfolios may increase the availability of different types of food for household consumption, in turn, improving the dietary quality of households [7,8,10,17,56]. Evidence from Zambia suggests a positive correlation between the number of crops cultivated and dietary diversity after controlling for household characteristics [17]. Sekabira et al. [8] find that increasing the level of agricultural production diversity significantly increases household diet diversity in Uganda. Oyarzun et al. [56] show agrobiodiversity to be a promising innovation pathway for improving the diet of smallholders in Ecuador.

The linkage between women’s empowerment and household outcomes has been explored in several studies [57,58,59,60,61]. Doss [57] shows that a woman’s share of assets, particularly farmland, significantly increases food budget shares in Ghana. In Cote d’Ivoire, Hoddinott et al. [58] suggest that increasing a woman’s share of the cash income significantly increases the share of the household budget allocated to food. Similarly, Sraboni et al. [59] illustrate that the greater empowerment of women, measured using the WEAI, increases diet diversity in Bangladesh. Ample evidence has shown that households do not act in a unitary manner when making decisions or allocating resources [60,61]. This means that men and women within households do not always have the same preferences or pool their resources. This creates a gender gap in the control of agricultural inputs, which has important implications for productivity and food security [62,63]. Three pathways are summarized to conceptualize the role of women in agriculture and the achieved nutritional outcomes, i.e., women’s social status and empowerment on their access to and control over resources, women’s participation in agriculture and their time allocation, and the health and nutritional status of women and families [64].

**Figure 1:**
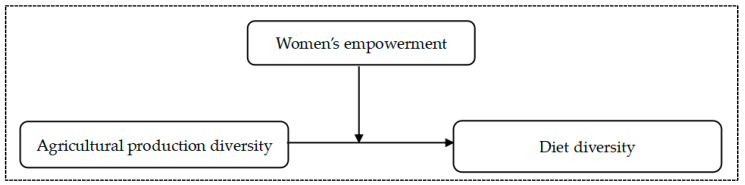
Conceptual framework diagram.

Drawing from the existing literature, we developed a conceptual framework for the role of women in mediating the causal relationship between agricultural production diversity and diet diversity for semi-subsistence households (see Figure 1). Using a household survey of food consumption that was conducted in China’s less developed areas in 2021, we tested the hypothesis by measuring women’s empowerment (WEI) and diet diversity (HDDS).

## 3. Materials and Methods

We used the quantitative data collected through a household survey organized by the Agricultural Information Institute, Chinese Academy of Agricultural Sciences (CAAS-AII). The survey of the present study was part of a larger research project on food security in China targeting regions for poverty reduction. Given the multiple purposes, a variety of modules were developed in questionnaires at the household and community levels. As the primary interest of the present study was households’ agricultural production and food consumption through gender-based perspectives, this research team developed specific modules on the topics. All study participants were asked for their informed verbal consent. This study used primary data without exposing any personal identification information.

### 3.1. Survey Design

**Figure 2:**
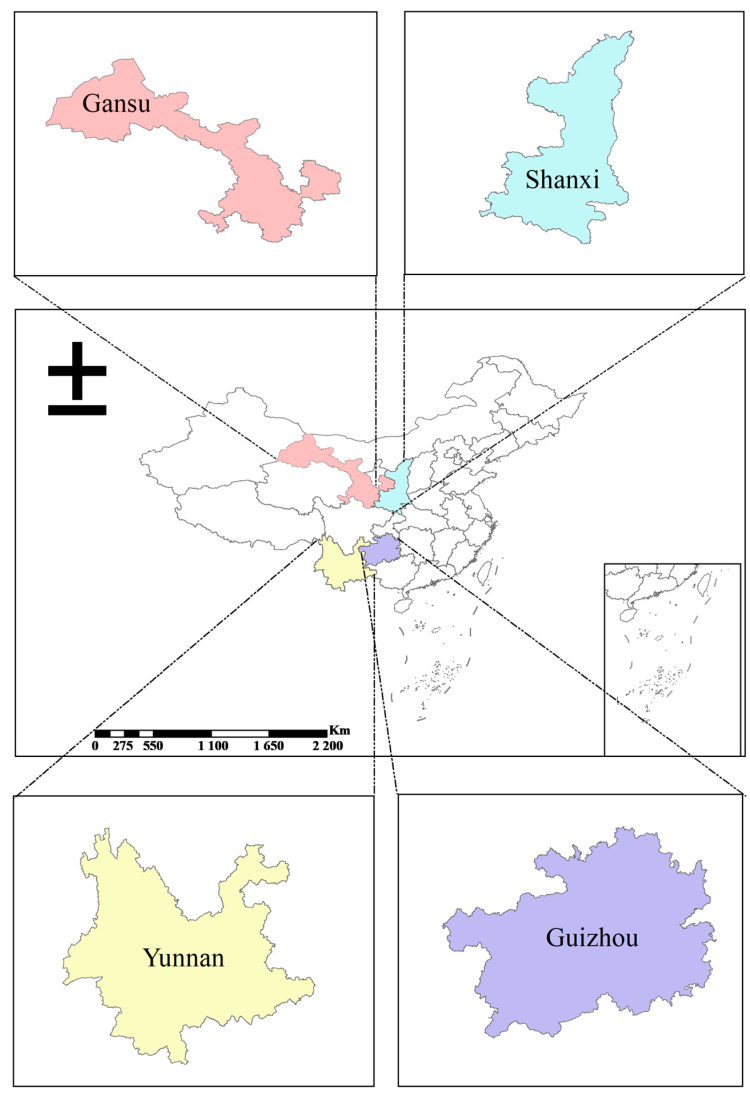
Map of sample districts for this study in China.

The study was conducted in less developed areas that were identified and targeted for China’s national actions of poverty reduction. The four surveyed provinces—Shaanxi, Yunnan, Guizhou, and Gansu—are all located in the western and southwestern parts of China (see Figure 2). These regions were targeted in China’s national campaign of poverty reduction [65]. The landscape of these areas is characterized by mountains and inland climates; in total, 78.9% of the terrain is mountainous. Agricultural production is mainly cultivated through drought-tolerant crops (such as corn, potatoes, beans, and vegetables) and raised livestock (pigs, chickens, and ducks). The degree of access to enabling markets was low, and the average distance to the nearest agricultural markets was 37.8 km for the sample villages. The per capita net income of the households was CNY 8899 in 2020, mainly from agricultural production. According to China’s 2022 poverty line (4000 CNY/year) standard, 7.92% of the region’s population was below the poverty line.

### 3.2. Sampling Methods

A combination of random and multi-stage samplings was used for the survey. First, the state of food security in 592 poverty-stricken counties was diagnosed by the national program of poverty reduction based on food availability, accessibility, and utilization; the counties were further divided into two categories of food security and food insecurity using cluster analysis. A total of 7 counties—Luonan County and Zhen’an County in Shaanxi Province, Qingshui County in Gansu Province, Wuding County and Huize County in Yunnan Province, and Panzhou City and Zheng’an County in Guizhou Province—were randomly selected from 271 counties where the state of food security was not performing [65]. Second, rural households were selected using the Probability Proportional Scale (PPS) sampling and random sampling methods. In the first stage, except for Qingshui County in Gansu Province, 19 of the populated administrative villages from each of the 6 sample counties were selected as the sample villages. Considering the availability of the data, 16 of the populated administrative villages from Qingshui County were selected. In the second stage, about 10 households were randomly selected from each sample village.

### 3.3. Data Collection

A questionnaire-based household survey was conducted between July and August 2021. The survey team consisted of a team of 10 researchers and 50 students. Data collectors received 3 days of training, including explaining the questionnaire content and the use of survey tools and simulation exercises. The survey was pilot-tested during data collectors training and an additional day of training after 3 days of data collection. Field teams, each consisting of 3–12 students depending on the size of the village, administered under a researcher. Data were collected using Android tablets. After sorting out uncompleted questionnaires, a total of 1199 household questionnaires were obtained in the 4 surveyed provinces. As shown in Table 1, about 36.5% of the interviewees were female, and the ratio was relatively higher for the provinces of Shanxi and Guizhou.

### 3.4. Data Analysis

Before analysis, the gathered data were prepared and cleaned. The dataset was checked for missing data and outliers. For this, the “outlier labeling rule” was used. All values outside the calculated range were considered outliers [66]. All statistical analyses were performed using the statistical software STATA v15.

Descriptive analysis was conducted to analyze the associations between women’s empowerment, dietary diversity, and agricultural production diversity. After exploring associations with women’s empowerment, dietary diversity, and agricultural production diversity, we used multivariate regressions (using a *p*-value < 0.10 to define significance) to control for individual characteristics and household-level attributes that may conjointly affect food consumption behavior, food security, as well as the choice of a coping strategy to quantify the correlation. Finally, for a continuous dependent variable, Ordinary Least Squares regression was used, and results from OLS regression were presented.

### 3.5. Construct of Key Variables

This study followed FAO’s FGIs method to measuring household dietary diversity and production diversity. Based on the households’ 24 h recall data, food consumption was categorized into twelve major food groups: cereals, stems, vegetables, fruits, meat, eggs, fish and seafood, milk, legumes, fats, sugars, and condiments. Then, the consumption frequency of each food group was computed based on whether they had been eaten in the 24 h period. In the calculation process, the same weighting was assigned to each food consumed, with each food assigned one point, while repeated consumption was not scored [16,56].

We measured agricultural production diversity by counting the different agricultural products, i.e., the number of crops grown and the livestock raised by farmers [11,67,68,69]. For each type of crop planted or livestock raised, one point was scored accordingly.

For robustness, the study used the Food Group Production Diversity Score (FGPD) to check the consistency of the model’s results [17,18]. The construction method of FGPD was similar to the HDDS. Agricultural products were categorized into nine food groups (cereals, stems, beans, vegetables, fruits, meat, fish, eggs, and milk). In the specific calculation process, the same weighting was assigned to each food group; production was assigned 1 point, and non-production was assigned a value of 0.

Based on the conceptual framework of agency and women’s empowerment, this study constructed a Women’s Empowerment Index (WEI) within the context of China [70]. In this study, women’s empowerment and agency comprised six indicators of five different domains. Table 2 shows a summary of women empowerment by domains, indicators, the level of adequacy in each indicator, and the corresponding weights.

This study used the Alkire–Foster counting approach to compute the Women’s Empowerment Index [71]. The methodology mainly measures women’s disempowerment in multiple dimensions. The index *M*_0_ was constructed across the dimensions of household decision making on production, resources, income, leadership, and time. The generation of the index took the following steps:

In step 1, all adequacy indicators in domains were first coded, assuming that the value was 1 if the individual lacked adequate achievements in that indicator and 0 otherwise. Then, an inadequacy score was computed for each person according to his or her inadequacies across all indicators.

In step 2, a cut-off regarding identifying who was disempowered was set, assuming equal weights for all dimensions. After exploring the sensitivity of the empowerment classification for the different cut-offs, we selected the disempowerment cut-off of 20% [71].

In step 3, the inadequacy score was censored. In a situation of less than or equal to the disempowerment cut-off (20%) for the inadequacy score, the score was replaced with 0.

In step 4, we computed the disempowerment index (*M*_0_). Specifically,

(1) $$H_{p}=\frac{q}{n}$$

where $H_{p}$ is the disempowered headcount ratio, *q* is the number of women who are disempowered, and *n* is the total population.

(2) $$A_{p}=\frac{\sum_{i=1}^{n}C_{i}(k)}{q}$$

where $A_{p}$ is the intensity of disempowerment, $C_{i}(k)$ is the censored inadequacy score, and *q* is the number of disempowered women.

(3) $$M_{0}=H_{p}\;*\;A_{p}$$

where $M_{0}$ is the disempowerment index.

(4) $$\mathrm{WEI}=1-M_{0}$$

where *WEI* is the Women’s Empowerment Index.

### 3.6. Econometric Modeling and Estimating Methods

When estimating the impacts of farmers’ production diversity on the household dietary diversity in China, we specified the basic model as follows:

(5) $$HDDS=\lambda_{0}+\lambda_{1}APD+\lambda_{2}WEMP+\lambda_{3}Controls+\eta$$

*HDDS* is a continuous variable of dietary diversity at the household level. The values ranged from zero to twelve on the basis of the number of consumed food groups. Agricultural production diversity (*APD*) measures the diversity level of agricultural production for a household; it is a continuous variable, taking a value between zero and nine. Women’s empowerment (*WEMP*) is a binary variable of women’s empowerment. A value of one was assigned when the inadequacy score was less than or equal to the dis-empowerment cut-off of 20%, representing a higher level of women’s empowerment; otherwise, a value of zero was assigned.

Based on the extant literature, it is likely that several other factors affect diet diversity. Consequently, we controlled, for example, additional characteristics of households, including the age of the household’s head, the education of the household’s head, household size, household wealth, the number of children (≤5 years old and 6–14 years old), the number of elderly people (≥65 years old), household income, expenditure, kitchen gardens, migrant workers, health status, credit, etc. Furthermore, community characteristics might vary from village to village and could affect household diet diversity. Therefore, we also included additional factors at the village level, such as market distance, the area of arable land, and major adverse weather event in the last 12 months.

We looked at the heterogenous effects since women’s empowerment may have affected sub-groups of production diversity in different ways. To examine these effects, we specified an extension of the basic model by interacting with the variables of *APD* and *WEMP*. The coefficient $\lambda_{3}$ measured the differential effect of production diversity on diet diversity for a stronger female agency relative to that of less women-empowered households. We paid particular attention to the sign of the coefficient on the interaction term; a positive coefficient indicated that women’s empowerment had a greater favorable impact on diet diversity with greater production diversity, and a negative coefficient implied that women’s empowerment mitigated the negative impacts of lower production diversity.

(6) $$HDDS=\lambda_{0}+\lambda_{1}APD+\lambda_{2}WEMP+\lambda_{3}APD\times WEMP+\lambda_{4}Controls+\eta$$

We estimated Equations (5) and (6) through the OLS model. 

## 4. Results and Discussion

### 4.1. Results

The diet diversity of the studied household seemed to correlate with production diversity. As illustrated in Table 3, the studied households were divided into three groups based on the levels of agricultural production diversity: Low Production Diversity (LPD) score ≤ two crop types, *n* = 414; Medium Production Diversity (MPD) score ≤ four crop types, *n* = 565; High Production Diversity (HPD) score > four crop types, *n* = 220. The HDDS of the LPD group households was 6.55, and the figures were significantly lower than the groups of production diversity at a higher level.

The mean of the HDDS in the high diet diversity group was 9.56, while the households consumed an average of 6.98 different food groups based on the 24 h recall period. Therefore, the mean of the HDDS in the HDD group was set as the target value to assess the household diet security following the FANTA. The diet diversity level of households in less developed areas needs to be further improved.

For the studied households in China’s less developed counties, the status of women’s empowerment was 0.689 (Table 4). In addition, the statistical results showed that the covariance of farmers’ diets and production diversity correlated with women’s empowerment. The WEI of the LPD and LDD group households was 0.618, and these figures were significantly lower than the group with LPD and HDD (0.694). Similarly, the WEI in the HPD group households increased from 0.685 in the LDD group to 0.694 in the HDD group. This suggests that empowering women can increase household diet diversity while the level of agricultural production diversity remains unchanged.

As shown in Table 5, agricultural production diversity has a direct benefit on household diet diversity. The coefficient on the variable of production diversity was positive at a 1% level of significance (β = 0.20), indicating the positive impact of production diversity on diet diversity. In particular, a unit increase in agricultural production diversity increased the likelihood of household diet diversity by 20% at a 1% level of significance. This implies that households that diversify their agricultural production are more likely to improve their diet quality.

The coefficient of women empowerment was positive and significant at a 5% level of significance (β = 0.23), demonstrating that a unit percent increase in women empowerment resulted in a 0.23 point increase diet diversity score. This suggests a strong and positive association between women’s empowerment and household diet diversity.

Table 5 also shows the heterogeneous effects of other control variables on household dietary diversity. The coefficient of the education level of household heads was positive and significant (β = 0.04), indicating a positive impact of education attainment on diet diversity. The estimate of the household wealth index reveals that diet diversity increases by 0.73 units if household wealth increases by 1 unit, and this implies that improving households’ wealth status helps increase the dietary quality of households. Similarly, the coefficient of the household income was significantly positive at a 1% level significance. It suggests that household income was positively correlated with the nutritional level of the household diet, i.e., an increase in household income by one percent point results in a 17% increase in diet diversity. The coefficient of expenditure on eating and drinking for going out was positive and significant (β = 0.11), which means that the higher the expenditure, the more likely the family members were exposed to diverse foods. In addition, the coefficient of market distance was significantly negative, demonstrating that the market distance had a significant negative impact on the household dietary diversity, and the closer the market distance, the more likely they reached the food market. The coefficient of land size implied that an increase in land size by one percent point resulted in an approximately 11% decrease in diet diversity of the household. However, other variables, e.g., the age of household head, household credit, household size, etc., had no significant effect on household diet diversity in the model.

Turning to the heterogeneous effects of gender, we could see strong evidence that women’s employment mitigated the side effects of decreasing production diversity on a household’s diet security. The coefficient of interaction term of production diversity and women’s empowerment was significantly negative (β = −0.19) at a 1% level of significance. This estimate indicated that empowering women can moderate the impact of low agricultural production diversity on household dietary diversity.

**Figure 3:**
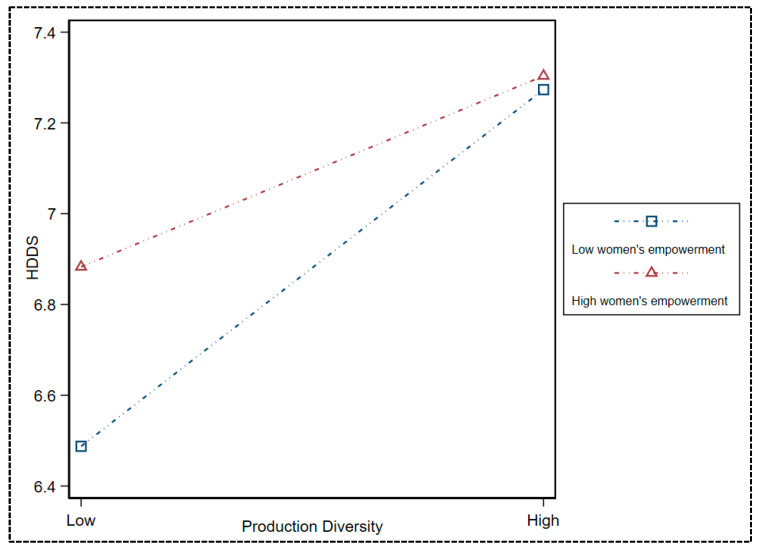
Interaction effect diagram.

The mitigating effects of women’s empowerment on a household’s diet diversity for a lower production diversity are visually displayed in Figure 3. The tilting slope of the line indicating low levels of women’s empowerment suggests that empowering women had a greater positive impact on household dietary diversity with low levels of production diversity than households with high levels of agricultural production diversity.

To diagnose the robustness of these main findings, we used the Food Group Production Diversity Score (FGPD) as an alternative measure of agricultural production diversity. The results in Table 6 are consistent with the findings of Table 5. The FGPD positively and significantly affected household diet diversity (β = 0.18), and women’s empowerment mitigated the negative effect of low agricultural production diversity on household diet security (β = −0.18). The results of the econometric analysis were robust across the use of different measures of production diversity.

### 4.2. Discussion

We chose to study less developed areas of China because low diversity in food consumption (dietary) remains a major problem for smallholder farmers in the region. Our results support the positive associations between agricultural production diversity and diet diversity at the household level, suggesting that policies of promoting production diversification can improve food and nutrition security. The findings are in line with the results of Huang et al. [6], who noted that, as other developing countries, agricultural production diversity in China promotes the households’ diets and nutritional health. It is also in line with Sekabira et al. [8] and Singh et al. [9], who reported that agricultural production diversity positively affects the food security and nutrition of household members. However, our estimations shows that, to increase the dietary diversity by one food group, the production diversity needs to increase by at least five food groups. Similar evidence was found by Sibhatu and Qaim [12], who reported that farmers in Sub-Saharan Africa would need to grow nine additional species to increase their dietary diversity by one food group. The integrative assessment of agricultural production diversity and the effects on labors and welfare is not yet conclusive.

Enabling market access in rural areas is a promising strategy to enhance dietary security and nutrition. The coefficients of the variable for market distance are significantly negative, suggesting a contribution of enabling market access to dietary diversity. Taking evidence from Indonesia, Kenya, Ethiopia, and Malawi, Sibhatu et al. [11] found that improving small farmers’ access to markets is more effective at improving nutrition than diversifying production for subsistence farmers. The enabling markets are not limited to agricultural produce; improved market access to chemical fertilizer was also found to be effective to improve dietary diversity in Malawi and Zambia [16,17]. When markets are fully liberalized, the potential gains in dietary diversity from further diversification become limited [18].

The findings of this study stress the essential role of women’s empowerment in reducing food and nutrition insecurity, even more so amidst low production diversity. In other words, women’s empowerment potentially mitigates the adverse consequences of low agricultural diversification on household diet quality. The results align with findings of an earlier study on women’s empowerment where it was found that women’s empowerment has a positive and significant effect on the women’s dietary diversity score regardless of technology adoption status in Kenya [22]. Ssennono et al. [23] found that depriving women of agency further dampens poverty by 8.7% from climatic shocks. The implication is that households with empowered women perform better to withstand the adversities of climatic shocks. As a result, integrating women’s empowerment interventions with agricultural policies can be more effective than relying solely on boosting agricultural production and improving the food quality and nutrition for households in less developed areas.

## 5. Conclusions

In this study, we analyzed the role of women’s empowerment in diversifying household diets and, thus, their contributions to food security and nutrition. We focused specifically on poverty-stricken regions in China and conducted a questionnaire-based household survey in 2021. Based on micro-level evidence from 1199 rural households, this study found a low score of female empowerment; the status of diet diversity, measured by the household dietary diversity score, varied by income and social class; and the average rate was low. There is strong evidence that women’s employment mitigates the side effects of decreasing production diversity on households’ diet security. As a result, women’s empowerment potentially mitigates the adverse consequences of low agricultural diversification of household diet quality in less developed areas.

Women working in rural areas are under highly unfavorable conditions, especially in less developed countries where alternative livelihoods are not available. Despite the importance of agrifood systems for women’s and their families’ well-being, women’s roles tend to be marginalized; women’s access to resources and enabling services continues to lag behind those of men. To bring about positive and lasting improvements in food security and nutritional outcomes, gender-transformative approaches highlight the changes in policies and social norms that are deeply rooted in the society. Despite great success in poverty reduction and women’s empowerment, this study finds varying nutritional outcomes that are measured by household dietary diversity in China’s “exiting” areas of poverty. For China’s rural revitalization strategy, the findings of the study caveat the focus of boosting agricultural productivity growth only and advocates the use of nutritional outcomes and healthy diets as the indicators. The development and adaptation of the WEI and HDDS in this study may potentially be useful for designing future projects of rural poverty in China and elsewhere. Our study adds value by realigning and repurposing pro-poor policies toward agrifood transformation that constitute a healthy diet and gender-responsive agrifood systems and provides a theoretical reference for solving problems of food insecurity and malnutrition.

This study has several limitations. The measure of dietary diversity was based on self-reported consumption in the previous days, which may by distorted by unusual intake and associated measurement error. Diets and food sources of rural households may vary throughout the different seasons of the year, depending on fluctuations in food availability and income. Such seasonality aspects are not considered in food analysis when using recall data of a single round for 2 days, as conducted in this study. Another limitation relates to the measurement of women’s empowerment and agency. For future research, the construct of women’s empowerment and agency needs to be adapted to a great diversity of local context. The availability and flexibility of optional add-on modules of the Women’s Empowerment Index would inspire the scaled-up use of these tools, allowing for the accountable monitoring and assessment of gendered interventions and policies in food and development areas.

## Figures and Tables

**Table 1:** Sample description of the study.

	County (*n*)	Village (*n*)	Household (*n*)	Female Interviewee (%)
Shanxi	2	38	345	43.5
Yunnan	2	38	372	32.3
Guizhou	2	38	327	35.9
Gansu	1	16	155	33.1
Total	7	130	1199	36.5

**Table 2:** Indicators for constructing the Women’s Empowerment Index (WEI).

Domain	Indicator	Definition of Adequacy	Weight
Production	Input in productive decisions	If the woman participates in an income-generating activity (either agricultural, non-agricultural, or both) individually and jointly	1/5
Resources	Ownership of assets	If the woman solely or jointly owns land, buildings, or vehicles	2/15
	Decisions on credit	If the woman participates in decision making concerning credit individually or jointly	1/15
Income	Control over use of income	If the woman participates in decisions regarding the use of income	1/5
Leadership	Group membership	If the woman is a member of at least one economic or social group	1/5
Time	Workload	If the woman works less than 10.5 h a day	1/5

**Table 3:** Household dietary diversity score (HDDS) by different levels of production diversity.

	Production Diversity			
	Total (N = 1199)	Low (N = 414)	Medium (N = 565)	High (N = 220)
Low Diet Diversity (N = 479)	5.21	5.07	5.33	5.34
Medium Diet Diversity (N = 479)	7.46	7.46	7.42	7.52
High Diet Diversity (N = 241)	9.56	9.56	9.53	9.61
HDDS (N = 1199)	6.98	6.55	7.12	7.43

**Table 4:** The Women’s Empowerment Index (WEI) at different levels of diet and production diversity.

	Production Diversity			
	Total (N = 1199)	Low (N = 414)	Medium (N = 565)	High (N = 220)
Low Diet Diversity (N = 479)	0.653	0.618	0.683	0.685
Medium Diet Diversity (N = 479)	0.729	0.744	0.727	0.715
High Diet Diversity (N = 241)	0.682	0.694	0.669	0.694
HDDS (N = 1199)	0.689	0.669	0.700	0.701

**Table 5:** Multiple regression analysis identifying the impact of agricultural production diversity and women’s empowerment on household dietary diversity score (HDDS).

		(1)	(2)	(3)	(4)	(5)
1	Production diversity (0 = Min; 9 = Max)	0.23 ***		0.16 ***	0.20 ***	0.20 ***
2	Women’s Empowerment (1 = Yes; 0 = No)		0.33 ***	0.93 ***	0.23 **	0.21 **
3	Interaction of production diversity and women’s empowerment					−0.19 ***
4	Control variables					
5	Age of household head (in years)				0.00	0.00
6	Education of household head (in years)				0.04 ***	0.04 ***
7	Household size (2 = Min; 12 = Max)				0.04	0.04
8	Wealth Index (0 = Min; 1 = Max) ^a^				0.72 ***	0.73 ***
9	Household borrowed money from friends and relatives in the past 12 months (1 = Yes; 0 = No)				0.20	0.21
10	Number of children ≤ 5 years old				0.17	0.16
11	Number of children 6–14 years old				−0.03	−0.03
12	Number of elderly ≥ 65 years old				−0.02	−0.03
13	Area of arable land (mu)				−0.11 **	−0.11 *
14	Household members received medical treatment in the past 12 months (1 = Yes; 0 = No)				−0.01	−0.01
15	Expenditure on eating and drinking for going out (CNY)				0.11 ***	0.11 ***
16	Household income (CNY 1000)				0.18 ***	0.17 ***
17	Migrant workers (1 = Yes; 0 = No)				0.01	−0.00
18	Has a backyard farm (1 = Yes; 0 = No) ^b^				−0.07	−0.06
19	Market distance (km)				−0.01 ***	−0.01 ***
20	Household suffered major adverse weather event in the past 12 months (1 = Yes; 0 = No)				0.02	0.03
21	Constant	6.26 ***	7.08 ***	6.55 ***	3.34 ***	4.00 ***

Note: ^a^ The Wealth Index (WI) was constructed using a multiple correspondence analysis performed on variables that coded for housing quality (type and size of house, number of persons per room, and floor, wall, and roof material) and facilities (electricity, source of drinking water, type of cooking fuel, and type of toilet facility), for assets (television, phone, mobile phone, refrigerator, radio, torch, and kerosene lamp), and for means of transport (automobile, bicycle, and motorcycle). For each household, the coordinate on the first axis of the correspondence analysis was interpreted as an index of the economic level; ^b^ these include cereals, fruits, vegetables, and pulses; * *p* < 0.1, ** *p* < 0.05, *** *p* < 0.01.

**Table 6:** Robustness test of the effect of agricultural production diversity and women’s empowerment on the household dietary diversity score (HDDS).

		(1)	(2)	(3)	(4)	(5)
1	FGPD (1 = Min; 9 = Max)	0.23 ***		0.14 ***	0.19 ***	0.18 ***
2	Women’s empowerment (1 = Yes; 0 = No)		0.33 ***	0.79 ***		0.25 **
3	Interaction of empowerment and FGPD			−0.19 **		−0.18 **
4	Control variables					
5	Age of household head (in years)				0.00	0.00
6	Education of household head (in years)				0.04 ***	0.04 ***
7	Household size (2 = Min; 12 = Max)				0.05	0.06
8	Wealth index (0 = Min; 1 = Max)				0.82 ***	0.82 ***
9	Household borrowed money from friends and relatives in the past 12 months (1 = Yes; 0 = No)				0.20	0.22 *
10	Number of children ≤ 5 years old				0.17	0.16
11	Number of children 6–14 years old				−0.04	−0.03
12	Number of elderly ≥ 65 years old				−0.04	−0.04
13	Household members received medical treatment in the past 12 months (1 = Yes; 0 = No)				−0.10 *	−0.08
14	Expenditure on eating and drinking for going out (CNY)				−0.02	−0.03
15	Area of arable land (mu)				0.11 ***	0.11 ***
16	Household income (CNY 1000)				0.17 ***	0.16 ***
17	Migrant workers (1 = Yes; 0 = No)				−0.02	−0.02
18	Has a backyard farm (1 = Yes; 0 = No)				−0.08	−0.07
19	Market distance (km)				−0.01 ***	−0.01 ***
20	Household suffered major adverse weather event in the past 12 months (1 = Yes; 0 = No)				0.04	0.04
21	Constant	6.33 ***	7.08 ***	3.43 ***	3.34	3.89 ***

Note: * *p* < 0.1, ** *p* < 0.05, *** *p* < 0.01.

## Data Availability

If the readers are interested in the research data of the article, they can contact the author for request at huangyanfang01@caas.cn.
